# Bi-allelic genetic variants in the translational GTPases GTPBP1 and GTPBP2 cause a distinct identical neurodevelopmental syndrome

**DOI:** 10.1016/j.ajhg.2023.11.012

**Published:** 2023-12-20

**Authors:** Vincenzo Salpietro, Reza Maroofian, Maha S. Zaki, Jamie Wangen, Andrea Ciolfi, Sabina Barresi, Stephanie Efthymiou, Angelique Lamaze, Gabriel N. Aughey, Fuad Al Mutairi, Aboulfazl Rad, Clarissa Rocca, Elisa Calì, Andrea Accogli, Federico Zara, Pasquale Striano, Majid Mojarrad, Huma Tariq, Edoardo Giacopuzzi, Jenny C. Taylor, Gabriela Oprea, Volha Skrahina, Khalil Ur Rehman, Marwa Abd Elmaksoud, Mahmoud Bassiony, Huda G. El Said, Mohamed S. Abdel-Hamid, Maha Al Shalan, Gohun Seo, Sohyun Kim, Hane Lee, Rin Khang, Mahmoud Y. Issa, Hasnaa M. Elbendary, Karima Rafat, Nikolaos M. Marinakis, Joanne Traeger-Synodinos, Athina Ververi, Mara Sourmpi, Atieh Eslahi, Farhad Khadivi Zand, Mehran Beiraghi Toosi, Meisam Babaei, Adam Jackson, SYNAPS Study Group, Michael G. Hannah, Enrico Bugiardini, Enrico Bertini, Yamna Kriouile, Mohamed El-Khorassani, Mhammed Aguennouz, Stanislav Groppa, Blagovesta M. Karashova, Jatinder S. Goraya, Tipu Sultan, Daniela Avdjieva, Hadil Kathom, Radka Tincheva, Selina Banu, Pierangelo Veggiotti, Alberto Verrotti, Marcello Lanari, Salvatore Savasta, Alfons Macaya, Barbara Garavaglia, Eugenia Borgione, Savvas Papacostas, Michail Vikelis, Viorica Chelban, Rauan Kaiyrzhanov, Andrea Cortese, Roisin Sullivan, Eleni Z. Papanicolaou, Efthymios Dardiotis, Shazia Maqbool, Shahnaz Ibrahim, Salman Kirmani, Nuzhat N. Rana, Osama Atawneh, Shen-Yang Lim, Gian V. Zuccotti, Gian L. Marseglia, Susanna Esposito, Farooq Shaikh, Paola Cogo, Giovanni Corsello, Salvatore Mangano, Rosaria Nardello, Donato Mangano, Annarita Scardamaglia, George Koutsis, Carmela Scuderi, Eugenia Borgione, Pietro Ferrara, Giovanna Morello, Massimo Zollo, Roberto Berni-Canani, Luigi M. Terracciano, Antonio Sisto, Sandra Di Fabio, Federica Strano, Giovanna Scorrano, Saverio Di Bella, Ludovica Di Francesco, Ganieva Manizha, Maksud Isrofilov, Ulviyya Guliyeva, Kamran Salayev, Samson Khachatryan, Georgia Xiromerisiou, Cleanthe Spanaki, Chiara Fiorillo, Michele Iacomino, Eugenio Gaudio, Francina Munell, Antonella Gagliano, Farida Jan, Roberto Chimenz, Eloisa Gitto, Lorenzo Iughetti, Gabriella Di Rosa, Mohamad Maghnie, Massimo Pettoello-Mantovani, Neerja Gupta, Madhulika Kabra, Hanene Benrhouma, Meriem Tazir, Gabriella Bottone, Giovanni Farello, Maurizio Delvecchio, Giulio Di-Donato, Makram Obeid, Sophia Bakhtadze, Nebal W. Saadi, Michele Miraglia-Del-Giudice, Rita Maccarone, Maha S. Zaki, Chahnez C. Triki, Majdi Kara, Ehsan G. Karimiani, Ahmed M. Salih, Luca A. Ramenghi, Marco Seri, Giovanna Di-Falco, Luana Mandarà, Giuseppe Barrano, Maurizio Elisa, Enrico Cherubini, Francesca F. Operto, Mariella Valenzise, Antonino Cattaneo, Francesca Zazzeroni, Edoardo Alesse, Sara Matricardi, Faisal Zafar, Ehsan Ullah, Erum Afzal, Fatima Rahman, Muhammad M. Ahmed, Pasquale Parisi, Alberto Spalice, Maria De Filippo, Amelia Licari, Edoardo Trebbi, Ferdinando Romano, Gali Heimer, Issam Al-Khawaja, Fuad Al-Mutairi, Fowzan S. Alkuraya, Mie Rizig, Chingiz Shashkin, Nazira Zharkynbekova, Kairgali Koneyev, Aida Bertoli-Avella, Alistair T. Pagnamenta, Marcello Niceta, Roberta Battini, Antonio Corsello, Chiara Leoni, Francesco Chiarelli, Bruno Dallapiccola, Eissa Ali Faqeih, Krishnaraya K. Tallur, Majid Alfadhel, Eman Alobeid, Sateesh Maddirevula, Kshitij Mankad, Siddharth Banka, Ehsan Ghayoor-Karimiani, Marco Tartaglia, Wendy K. Chung, Rachel Green, Fowzan S. Alkuraya, James E.C. Jepson, Henry Houlden

**Affiliations:** 1 Department of Neuromuscular Diseases, UCL Queen Square Institute of Neurology, London, UK; 2 Department of Clinical Genetics, Human Genetics and Genome Research Institute, National Research Centre, Cairo, Egypt; 3 Howard Hughes Medical Institute, Department of Molecular Biology and Genetics, Johns Hopkins University School of Medicine, Baltimore, MD 21205, USA; 4 Molecular Genetics and Functional Genomics, Ospedale Pediatrico Bambino Gesù, IRCCS, 00146 Rome, Italy; 5 Department of Clinical and Experimental Epilepsy, UCL Queen Square Institute of Neurology, London, UK; 6 Institute of Neuro- and Behavioral Biology, Westfälische Wilhelms University, Münster, Germany; 7 Genetic and Precision Medicine Department, King Abdullah Specialized Children Hospital, King Abdulaziz Medical City, Ministry of National Guard Health Affairs (MNGHA), Riyadh, Saudi Arabia; 8 King Abdullah International Medical Research Center (KAIMRC), King Saud bin Abdulaziz University for Health Sciences, Ministry of National Guard Health Affairs (MNGHA), Riyadh, Saudi Arabia; 9 Arcensus GmbH, Rostock, Germany; 10 Division of Medical Genetics, Department of Pediatrics, McGill University, Montreal, Canada; 11 Unit of Medical Genetics, IRCCS Istituto Giannina Gaslini, Genoa, Italy; 12 Department of Neurosciences, Rehabilitation, Ophthalmology, Genetics, Maternal and Child Health, University of Genoa, Genoa, Italy; 13 Unit of Pediatric Neurology, IRCCS Istituto Giannina Gaslini, Genoa, Italy; 14 Department of Medical Genetics, Faculty of Medicine, Mashhad University of Medical Sciences, Mashhad, Iran; 15 Health Biotechnology Division, National Institute for Biotechnology and Genetic Engineering (NIBGE), Faisalabad, Pakistan; 16 National Institute for Health Research Oxford Biomedical Research Centre, Oxford, UK; 17 Genomics Research Centre, Human Technopole, Milan, Italy; 18 Wellcome Centre for Human Genetics, University of Oxford, Oxford OX3 7BN, UK; 19 Town Women and Children Hospital, Peshawar, Pakistan; 20 Neurology Unit, Department of Pediatrics, Faculty of Medicine, Alexandria University, Alexandria, Egypt; 21 Faculty of Medicine, University of Alexandria, Alexandria, Egypt; 22 Department of Family Health, High Institute of Public Health, University of Alexandria, Alexandria, Egypt; 23 Department of Medical Molecular Genetics, Human Genetics and Genome Research Institute, National Research Centre, Cairo, Egypt; 24 3billion, Inc, Seoul, South Korea; 25 Laboratory of Medical Genetics, St. Sophia’s Children’s Hospital, National and Kapodistrian University of Athens, Athens, Greece; 26 Genetics Unit, Department of Obstetrics & Gynaecology, Aristotle University of Thessaloniki, Papageorgiou General Hospital, Thessaloniki, Greece; 27 Paediatric Outpatient Clinic, Xanthi, Greece; 28 Department of Medical Genetics, Faculty of Medicine, Mashhad University of Medical Sciences, Masshad, Iran; 29 Student Research Committee, Faculty of Medicine, Mashhad University of Medical Sciences, Masshad, Iran; 30 Mashhad Genetic Counselling Center, Masshad, Iran; 31 Pediatric Neurology Department, Ghaem Hospital, Mashhad University of Medical Sciences, Mashhad, Iran; 32 Department of Pediatrics, North Khorasan University of Medical Sciences, Bojnurd, Iran; 33 Manchester Centre for Genomic Medicine, St Mary’s Hospital, Manchester University NHS Foundation Trust, Health Innovation Manchester, Manchester M13 9WL, UK; 34 CENTOGENE GmbH, Rostock, Germany; 35 Department of Developmental Neuroscience, IRCCS Stella Maris Foundation, 56128 Pisa, Italy; 36 Department of Clinical and Experimental Medicine, University of Pisa, 56126 Pisa, Italy; 37 Department of Clinical Sciences and Community Health, University of Milan, Milan, Italy; 38 Center for Rare Diseases and Birth Defects, Department of Women and Child Health and Public Health, Fondazione Policlinico Universitario A. Gemelli IRCCS, 00168 Rome, Italy; 39 Department of Pediatrics, University of Chieti, 66100 Chieti, Italy; 40 Unit of Medical Genetics, Children’s Specialist Hospital, King Fahad Medical City, Riyadh, Saudi Arabia; 41 Royal Hospital for Sick Children, Edinburgh, UK; 42 College of Medicine, King Saud Bin Abdulaziz University for Health Sciences, Ministry of National Guard Health Affairs (MNGH), Riyadh, Saudi Arabia; 43 Department of Genetics, King Faisal Specialist Hospital and Research Center, Riyadh, Saudi Arabia; 44 Department of Neuroradiology, Great Ormond Street Hospital, London, UK; 45 Genetics Research Centre, Molecular and Clinical Sciences Institute, University of London, St George’s, Cranmer Terrace, London SW17 0RE, UK; 46 Department of Pediatrics, Boston Children’s Hospital Harvard Medical School, Boston, MA 02115, USA

**Keywords:** neurodevelopmental disorders, neurodegeneration, NBIA, ribosomopathies, ribosome stalling, GTPBP1, GTPBP2, animal models, ectodermal disorders, GREND syndrome

## Abstract

The homologous genes *GTPBP1* and *GTPBP2* encode GTP-binding proteins 1 and 2, which are involved in ribosomal homeostasis. Pathogenic variants in *GTPBP2* were recently shown to be an ultra-rare cause of neurodegenerative or neurodevelopmental disorders (NDDs). Until now, no human phenotype has been linked to *GTPBP1*. Here, we describe individuals carrying bi-allelic *GTPBP1* variants that display an identical phenotype with *GTPBP2* and characterize the overall spectrum of GTP-binding protein (1/2)-related disorders. In this study, 20 individuals from 16 families with distinct NDDs and syndromic facial features were investigated by whole-exome (WES) or whole-genome (WGS) sequencing. To assess the functional impact of the identified genetic variants, semi-quantitative PCR, western blot, and ribosome profiling assays were performed in fibroblasts from affected individuals. We also investigated the effect of reducing expression of *CG2017*, an ortholog of human *GTPBP1/2*, in the fruit fly *Drosophila melanogaster*. Individuals with bi-allelic *GTPBP1* or *GTPBP2* variants presented with microcephaly, profound neurodevelopmental impairment, pathognomonic craniofacial features, and ectodermal defects. Abnormal vision and/or hearing, progressive spasticity, choreoathetoid movements, refractory epilepsy, and brain atrophy were part of the core phenotype of this syndrome. Cell line studies identified a loss-of-function (LoF) impact of the disease-associated variants but no significant abnormalities on ribosome profiling. Reduced expression of *CG2017* isoforms was associated with locomotor impairment in *Drosophila*. In conclusion, bi-allelic *GTPBP1* and *GTPBP2* LoF variants cause an identical, distinct neurodevelopmental syndrome. Mutant *CG2017* knockout flies display motor impairment, highlighting the conserved role for GTP-binding proteins in CNS development across species.

## Graphical abstract

**Figure:**
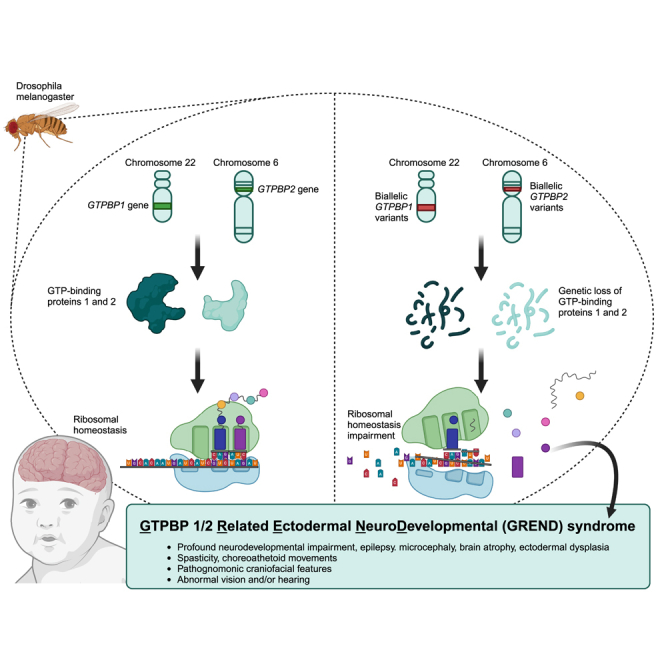

## Teaser

Bi-allelic variants in the translational GTPases GTPBP1 and GTPBP2 may affect ribosomal translational control and impair brain development and neurological function. This research identifies 20 individuals with homozygous GTPBP1 and GTPBP2 variants leading to an identical neurodevelopmental syndrome, which we defined as “Gtpbp1/2-related ectodermal neurodevelopmental (GREND) syndrome.”

## Introduction

*GTPBP1* (MIM: 602245) and *GTPBP2* (MIM: 607434) encode GTP (guanosine triphosphate)-binding proteins 1 and 2 (GTPBP1 and GTPBP2), which share 68% sequence similarity. These proteins are involved in many aspects of ribosomal homeostasis and mRNA translation, but their role in the development of the central nervous system (CNS) is still poorly characterized.^1^^,^^2^^,^^3^

Rare genetic variants in *GTPBP2* have been reported in recent years as a very rare cause of neurodegeneration with brain iron accumulation (NBIA) and/or severe neurodevelopmental disorders in a small group of consanguineous families from Iran, Northern Africa, and the Middle East.^4^^,^^5^^,^^6^^,^^7^ However, limited genotype-phenotype correlations are available for this ultra-rare condition, and the underlying disease mechanisms remain largely elusive. In addition, there is no yet established human disease associated with the homologous *GTPBP1*.

In this study, we report 20 individuals from 16 families carrying bi-allelic variants in either *GTPBP1* or *GTPBP2*, displaying a distinctive and recognizable syndromic neurodevelopmental condition. Phenotypic analysis of affected individuals refines the clinical presentations previously associated with *GTPBP2-*related NDDs and also highlights a similar overlapping syndrome associated with bi-allelic variants in either *GTPBP1* or *GTPBP2*. Here, we define the core phenotype associated with bi-allelic genetic defects in translational GTPases GTPBP1 and GTPBP2.

The impact of the identified genetic variants on mRNA and protein levels, as well as on translational efficiency, was investigated on fibroblasts from affected individuals. *Drosophila* carrying hypomorphic variants in a *GTPBP1*/*GTPBP2* ortholog, *CG2017*, were also evaluated for motor defects, complementing evidence on the conserved role of translational GTPases in CNS development from previous animal model (mice) studies.

## Material and methods

### Recruitment of research subjects

Families with undiagnosed NDDs were recruited from England, Italy, Greece, Libya, Saudi Arabia, Egypt, Pakistan, and Iran through international research networks as well as using GeneMatcher (www.genematcher.org).^8^ Informed consent for DNA analysis was obtained from study participants in line with local institutional review board requirements at the time of collection. Individuals (and/or their legal guardians) recruited in this study gave informed consent for their research participation. Those individual research studies received approval from the Review Boards and Bioethics Committees at University College London Hospital (project 06/N076) and the other institutions involved in this study. The procedures followed in this study were in accordance with the ethical standards of the responsible committees on human experimentation. Permission for inclusion of their anonymized medical data in this cohort, including photographs, was obtained using standard forms at each local site by the responsible referring physicians. All variations identified by whole-exome sequencing (WES) or whole-genome sequencing (WGS) were confirmed by Sanger sequencing. In all families, available unaffected parents and relatives were tested for the identified variants by targeted Sanger sequencing. For each affected individual, clinical data, as well as brain imaging and electroencephalography (EEG), were reviewed by pediatric neurologists, medical geneticists, and pediatric neuroradiologists from the participating centers.

### Genetic and molecular studies

Single-nucleotide variations (SNVs) were identified by WES or WGS in all individuals of this cohort. Genomic DNA was extracted from the whole blood or saliva of the affected individuals and their parents. Exomes or genomes were captured and sequenced on Illumina sequencers. Raw data were processed and filtered with established pipelines at diagnostic or research laboratories as described previously.^9^^,^^10^^,^^11^^,^^12^ Variant (single nucleotide and indel) calling and filtering was performed using the Genome Analysis Tool Kit (GATK). Variants that did not adhere to the following criteria were excluded from further analysis: allele balance of >0.70, QUAL of >20, and coverage of >20×. Rare variations present at a frequency above 1% in gnomAD (https://gnomad.broadinstitute.org/) or present from exomes or genomes within datasets from UK Biobank and the UK 100,000 Genomes Project or from internal research databases (e.g., Queen Square Genomics and UCL SYNaPS Study Group) were excluded. Candidate variants were then inspected with the Integrative Genomics Viewer and then confirmed by Sanger sequencing in all the families and submitted to the Leiden Open Variation Database (LOVD; https://www.lovd.nl/3.0/home).

### Sequence variant interpretation and expression studies

Sequence variants in *GTPBP1* or *GTPBP2* are numbered based on the reference sequences GenBank: NM_004286 and GenBank: NM_016373, respectively. Description of the sequence (Human Genome Variation Society) was done with the assistance of Mutalyser v.2.0.26.^13^ Sequence candidate variants were interpreted according to the ACMG Guidelines.^14^ Also, we explored the regional expression of *GTPBP1* or *GTPBP2* in the normal human brain. We used microarray data (Affymetrix Exon 1.0 ST) from human postmortem brain tissue collected by the UK Human Brain Expression Consortium as previously described.^15^

### Functional studies

To investigate the functional impact of the identified variants, we performed a reverse-transcriptase polymerase chain reaction (RT-PCR), and western blots (WBs) were performed in available fibroblast cell lines from consenting families. Semi-quantitative PCR (semi-qPCR) was performed in 50-μL reaction volume prepared by combining the cDNA template, gene-specific primers (see supplemental information), nuclease-free water, and SYBR Green Master Mix. The PCR reaction conditions are reported in the supplemental information. In semi-qPCR experiments, all measurements were made in triplicate, and GAPDH was used as an endogenous reference gene, with amplification under the same conditions. The PCR products were then loaded in a 1% agarose gel, and densitometry analysis was carried out. For western blot analysis, protein lysates were obtained from cultured fibroblasts, and total protein concentration was measured by means of the Bradford assay. Samples were separated on SDS-PAGE using Bis-Tris gradient gels (4%–12% NuPAGE, Invitrogen) according to the manufacturer’s recommendations and electrophoretically transferred into Immobilon-P transfer membranes (Millipore). Membranes were immunoblotted with the antibody at 4°C overnight (see supplemental information). Blots were then exposed to horseradish peroxidase-conjugated goat anti-mouse IgG (1706516, Bio-Rad Laboratories, 1:5,000) for 1 h at room temperature. Blots were developed using ECL-Prime (GE Healthcare), visualized via a ChemiDoc Touch Imaging System, and analyzed using Image Lab v.5.2 software (Bio-Rad Laboratories). For the quantifications, the signal intensity of the affected protein bands was normalized to the signal intensity of GAPDH bands.

### Ribosome profiling experiments

Ribosome profiling samples were prepared as previously reported^16^ with several modifications (see supplemental information). Sequencing libraries were prepared by digesting 20 μg of RNA with 750 U of RNase I (Ambion) for 1 h at 25°C, yielding ribosome protected fragments (RPFs). Digestions were stopped with 10 μL of SUPERase^∗^In (Thermo Fisher Scientific), and ribosomes were pelleted through a 1 M sucrose cushion at 100,000 rpm, 4°C, for 1 h in a TLA 100.3 rotor using a Beckmann-Coulter Optima MAX ultracentrifuge. RPFs were extracted using the miRNeasy mini kit (Qiagen) and size-selected by denaturing PAGE using a 15% TBE-Urea gel. RNA fragments corresponding to 15–35 nucleotides were excised, dephosphorylated using T4 PNK (New England Biolabs), and ligated for 3 h at 37°C using T4 RNA ligase 2, truncated (New England Biolabs) to a 3′ oligonucleotide adapter with a unique molecular identifier (UMI) hexanucleotide degenerate sequence. Ribosomal RNA was depleted using RiboZero (Illumina), omitting the final 50°C incubation step. RNA was reverse-transcribed using Superscript III (New England Biolabs) using real-time primer with a second four-nucleotide UMI sequence, RNNN. cDNA was then circularized using circLigase (Lucigen) and amplified by PCR using Phusion high-fidelity polymerase (New England Biolabs). PCR-amplified libraries were quantified with a Bioanalyzer 2100 (Agilent) using the High-Sensitivity DNA kit and sequenced using a HiSeq2500 (Illumina) at the Johns Hopkins Institute of Genetic Medicine. Ribosome profiling data were then analyzed as previously reported.^17^^,^^18^

### *Drosophila* studies

*Drosophila* stocks were obtained from the Bloomington *Drosophila* Stock Center (see supplemental information). The *CG2017* (0758-G4) and *CG2017* (0269-G4) stocks denote two independent piggyBac transposable element insertions into the 5′ UTR region of the *CG2017* locus encoding isoform C (https://flybase.org/reports/FBgn0037391). These insertions were outcrossed into an isogenic iso31 background for five generations prior to experiments to control for potential differences in genetic background. As a paired control stock, we used the iso31 strain. Experiments involving trans-heterozygous combinations of *CG2017* (0758-G4) and *CG2017* (0269-G4) were performed using non-outcrossed insertions. *CG2017* is predicted to be the closest fly ortholog of *GTPBP2* by 13 of 14 possible alignment algorithms (https://flybase.org/reports/FBgn0037391) and is also highly orthologous to *GTPBP1*. We note that the *Drosophila* genome also contains a *CG2017* paralog (*Dgp-1*) not studied herein that is more closely related to *GTPBP1* than *CG2017*. To determine the impact of the above piggyBac elements on CG2017 isoform C expression, we utilized quantitative PCR (qPCR). Total RNA was extracted from ∼10–20 fly heads using TRIzol reagents, following manufacturer’s instructions (Thermo Fisher Scientific). Reverse transcription and qPCR reactions were performed using standard protocols. RNA was treated with DnaseI to remove contaminating gDNA (New England Biolabs, M0303S) and converted to cDNA using MMLV-RT (Promega, M170A). qPCR reactions were performed using the Power SYBR Green Master Mix (Thermo Fisher Scientific). Primers used for qPCR reactions are detailed in supplemental information. PCR reactions were performed in a 96-well Applied Biosystems Step One module using standard thermocycle protocols. Confocal imaging was performed using an inverted Zeiss 710 confocal microscope. Adult male *Drosophila* brains were dissected and immuno-stained as described previously.^19^ Primary and secondary antibodies used are listed in supplemental information. Locomotor activity was quantified using the *Drosophila* Activity Monitor (DAM) system (Trikinetics Inc.), in which activity is measured via breaking of a central infra-red beam.^20^ For DAM experiments, 3- to 5-day-old adult male *Drosophila* were loaded into glass tubes containing a 4% sucrose and 2% agar food source, and activity was recorded under 12 light:12 dark (12L:12D) cycles at 22°C–25°C.

## Results

We report molecular, clinical, and cellular findings in 20 affected individuals from 16 families affected with severe neurodevelopmental disorders and bi-allelic variants in *GTPBP1* or *GTPBP2*.

### Molecular findings

We identified nonsense (n = 2) and missense (n = 1) homozygous variants in *GTPBP1* (GenBank: NM_004286) and nonsense (n = 5), missense (n = 4), splicing (n = 2), and frameshift (n = 2) homozygous variations in *GTPBP2* (GenBank: NM_019096.5) (Table S1). The missense variants were classified as damaging by SIFT, PolyPhen-2, and Mutation Taster, with an average CADD score of 25. None of the variants identified were present in the homozygous state in gnomAD (Table S1).

### Demographic data

**Figure 1:**
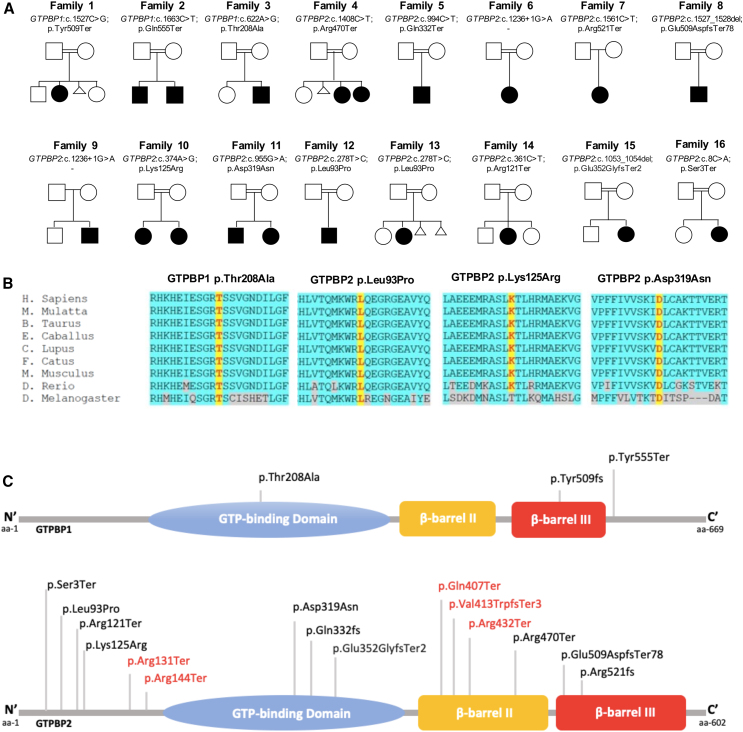
Genetic summary of the reported individuals with homozygous *GTPBP1* and *GTPBP2* variants (A) Pedigrees of the sixteen families described. Square, male; circle, female; black filled symbol, affected individual; white symbols, unaffected individuals. Double lines indicate consanguinity. (B) Protein multiple sequence alignment in *GTPBP1* and *GTPBP2* orthologs shows high conservation of the residues involved by the non-synonymous variants (highlighted in yellow) and almost complete conservation of the nearby residues (highlighted in light blue). (C) Schematic diagram indicating the domains of the GTPBP1 and GTPBP2 proteins, containing 669 and 602 amino acid residues, respectively. The orange shape represents the GTP-binding domain. The green and yellow boxes indicate the two beta-barrel domains. Variants reported in this study are represented in black, while previously reported variants in red.

Twenty individuals (from 16 families) were identified with homozygous variants either in *GTPBP1* (n = 4) or *GTPBP2* (n = 16). Nine of them were male (45%) and 11 were female (55%). The median age at last follow-up was 5.3 years. Among the 16 families, 15 were consanguineous (Figure 1; Table S2).

### A distinct clinical and imaging phenotype result from genetic defects either in GTPBP1 or GTPBP2

**Figure 2:**
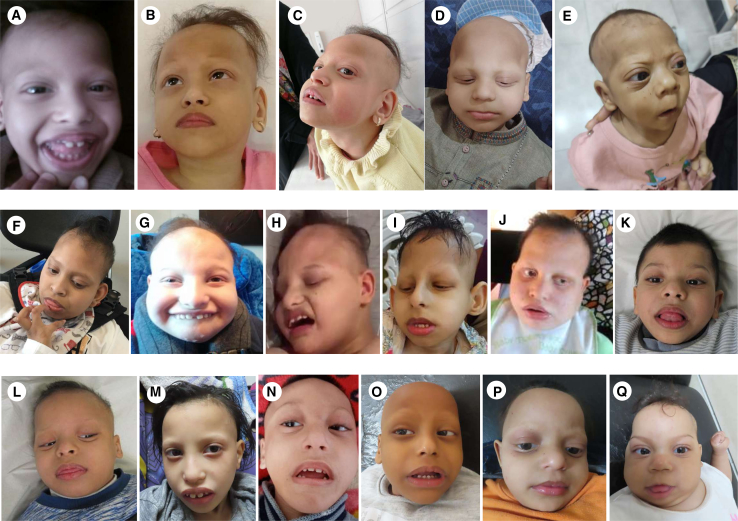
Distinctive craniofacial features associated with *GTPBP1/2*-related disorders Facial pictures of I-1 (A–C), I-2 (D), I-3 (E), I-5 (F), I-7 (G and H), I-8 (I), I-9 (J), I-10 (K), I-11 (L), I-12 (M), I-14 (N), I-16 (O), I-17 (P), and I-18 (Q). Note the distinct phenotype shared by individuals with variants in *GTPBP1* and *GTPBP2*, including microcephaly, high frontal hairline, sparse eyebrows and scalp hair, prominent nasal bridge, deep-set eyes, full lips, full cheeks, and abnormal dentition.

**Figure 3:**
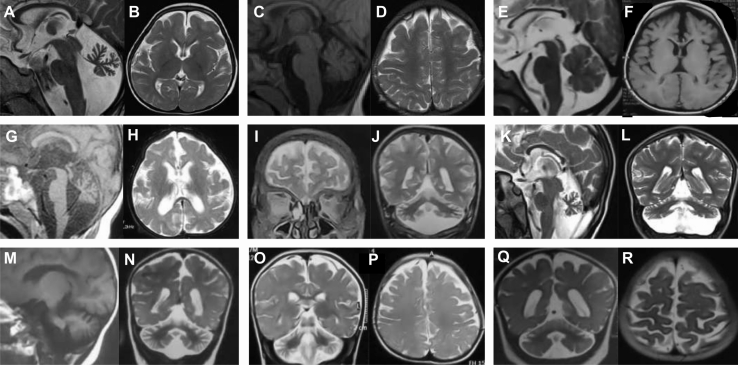
Brain imaging features associated with *GTPBP1/2*-related disorders Montage of cross-sectional MR images representing the spectrum of findings on neuroimaging. I-1 (A–D), I-4 (E and F), I-9 (G and H), I-12 (I and J), I-14 (K and L), I-16 (M and N), I-17 (O and P), I-18 (Q and R). The primary abnormalities included global cerebellar hypoplasia and atrophy (I-1, I-4, I-5, I-6, I-7, I-8, I-9), cerebellar hypoplasia (I-2, I-3), callosal hypoplasia (I-3, I-4, I-6), and underdevelopment and/or atrophy of the frontotemporal regions with involvement of the opercula (all individuals).

Congenital or early postnatal microcephaly was observed in all affected individuals. Mean occipitofrontal circumference (OFC) at birth was −1.4 SD (standard deviation), and 4 of 20 (20%) individuals presented postnatal microcephaly (defined by OFC ≤ −3 SD). Mean height was −1.1 SD, with only one individual under 3 SD. Distinctive facial features were reported in all affected individuals, variably including a coarse face, high temporal and frontal hairline, bi-temporal narrowing, full cheeks, thick protruding lips, and macroglossia (Figures 2, S1, and S2). Hand and/or feet anomalies were common (13/20; 65%) and included proximal (interphalangeal) contractures and congenital deformities such as tapered fingers or *pes cavus* (Figure S3). Distinct ectodermal features were variably present in all affected individuals, including sparse brittle hair, sparse eyebrows and eyelashes, abnormal skin pigmentation, and abnormal dentition (Figures 2, S1, and S2). Feeding difficulties were reported in 8 of 20 (40%) individuals. All affected individuals had profound neurodevelopmental impairment and were not able to sit (without support) or to walk. Axial hypotonia was observed in all affected individuals since the first months of life. All affected individuals were diagnosed with severe to profound intellectual disability (ID) and absent speech. Abnormal movements starting in infancy were reported in 15 of 20 (75%) individuals. These included stereotyped hand movements and (predominantly distal) choreoathetoid movements typically involving the hands (Videos S1, S2, S3, S4, and S5). Severe progressive spasticity (higher in the lower limbs than in the upper limbs) was observed in 17 of 20 affected individuals (85%; Figure S2). In total, 10 of 20 individuals developed seizures (50%). Where onset of seizures was reported, this was in the first year of life. Seizures were of varying types (including tonic, tonic-clonic, and myoclonic), either focal or generalized; however, a defined epileptic syndrome (based on clinical and EEG presentation) did not emerge. Drug resistance was reported in 3 out of 10 affected individuals (33%). Abnormal hearing and/or vision were reported in most individuals (14/20; 70%). Affected individuals had poor or absent eye contact since the first months of life, and electrodiagnostic tests were abnormal in 4 individuals that were found with retinal dysfunction (abnormal flash response) on electroretinogram (Table S2). Brain magnetic resonance imaging (MRI), where available, was abnormal in all affected individuals, and these studies showed global cerebellar atrophy with hypoplasia (I-1, I-4, I-5, I-6, I-7, I-8, I-9; Table S2), cerebellar hypoplasia (I-2, I-3; Table S1), callosal hypoplasia (I-3, I-4, I-6; Table S2), and underdevelopment and/or atrophy of the frontotemporal regions (with involvement of the opercula) in all affected individuals (Figure 3). Consistent with clinical and imaging phenotypes of *GTPBP1*- and *GTPBP2*-variant carriers, expression of these genes was found to be significantly enriched in the putamen and cerebellum, respectively (Figures S4 and S5).

Video S1. Individual I-1 (family 1) carrying the p.Tyr509Ter homozygous GTPBP1 variant Note the distinct craniofacial features (e.g., microcephaly, high frontal hairline, sparse eyebrows and hair, prominent nasal bridge, full lips, full cheeks, abnormal dentition), spastic tetraparesis (lower limbs > upper limbs), and hyperkinetic distal choreoathetoid movements.

Video S2. Individual I-5 (family 4) carrying the p.Arg470Ter homozygous GTPBP2 variant Note the distinct craniofacial features (e.g., microcephaly, high frontal hairline, sparse eyebrows and hair, full lips, full cheeks, abnormal dentition), spastic tetraparesis, poor spontaneous motility, and peculiar athetoid distal (hand) movements.

Video S3. Individual I-6 (family 5) carrying the p.Gln332Ter homozygous GTPBP2 variant Note the distinct craniofacial features (e.g., microcephaly, high frontal hairline, sparse eyebrows and hair, full lips, full cheeks, abnormal dentition), protruded tongue, yellowish skin, spastic tetraparesis, and peculiar athetoid distal (hand) movements.

Video S4. Individual I-9 (family 7) carrying the p.Arg521Ter homozygous GTPBP2 variant Note the distinct craniofacial features (e.g., microcephaly, high frontal hairline, sparse eyebrows and hair, full lips, full cheeks, abnormal dentition), protruded tongue, spastic tetraparesis, very poor spontaneous motility, and peculiar athetoid distal (hand) movements.

Video S5. Individual I-10 (family 8) carrying the p.Glu509Ter homozygous GTPBP2 variant Note the distinct craniofacial features (e.g., microcephaly, high frontal hairline, sparse eyebrows, full lips, full cheeks, abnormal dentition), protruded tongue, spastic tetraparesis, and hyperkinetic athetoid distal (hand) movements.

### RT-PCR and western blot highlight a loss-of-function mechanism

Reverse transcription PCR amplifying the variant cDNA transcript from mRNA extracted from available fibroblast cell lines of *GTPBP2* variant carriers identified a significant reduction of mRNA compared to the age-matched control (Figure S6), as confirmed by analysis of semi-qPCR using the densitometry software ImageJ after normalization relative to a housekeeping gene (GAPDH) and calculation using a relative relationship method (Figure S6). The characterization of the splicing variant showed intron retention (second lane; 365 bp) products as a result of the splicing homozygous variant, compared to the positions of correctly spliced *GTPBP2* (first lane; 228 bp) and intron retention (second lane; 365 bp) (Figure S6). Western blot showed a significant reduction of the GTPBP2 protein in fibroblasts from affected individuals compared to cell lines from healthy controls (Figure S6).

### Translational efficiency is not significantly impaired in cell lines from affected individuals

To investigate potential defects in translation arising from genetic loss of GTPBP2, we employed ribosome profiling^16^ in fibroblasts derived from a healthy control as well as from available individuals carrying the homozygous p.Gln332Ter and c.1236+1G>A variants in *GTPBP2* (GenBank: NM_019096.6). Ribosome density on protein coding transcripts (Figure S7-A, Pearson’s r > 0.97) showed that translation was not broadly perturbed in these cell lines. To test the role of GTPBP2 in rescuing stalled ribosomes, we evaluated footprint size distributions (Figure S7B) and relative codon occupancies (Figure S7C). We observed no global enrichment or codon-specific enrichment of stalled ribosomes with empty A sites corresponding to 21 nucleotide reads^3^ in GTPBP2-deficient cells. Taken together, we did not observe a significant translation defect in fibroblast cell lines from affected individuals.

### Suppressing expression of the *GTPBP1/2* ortholog *CG2017* reduces locomotor activity in *Drosophila*

**Figure 4:**
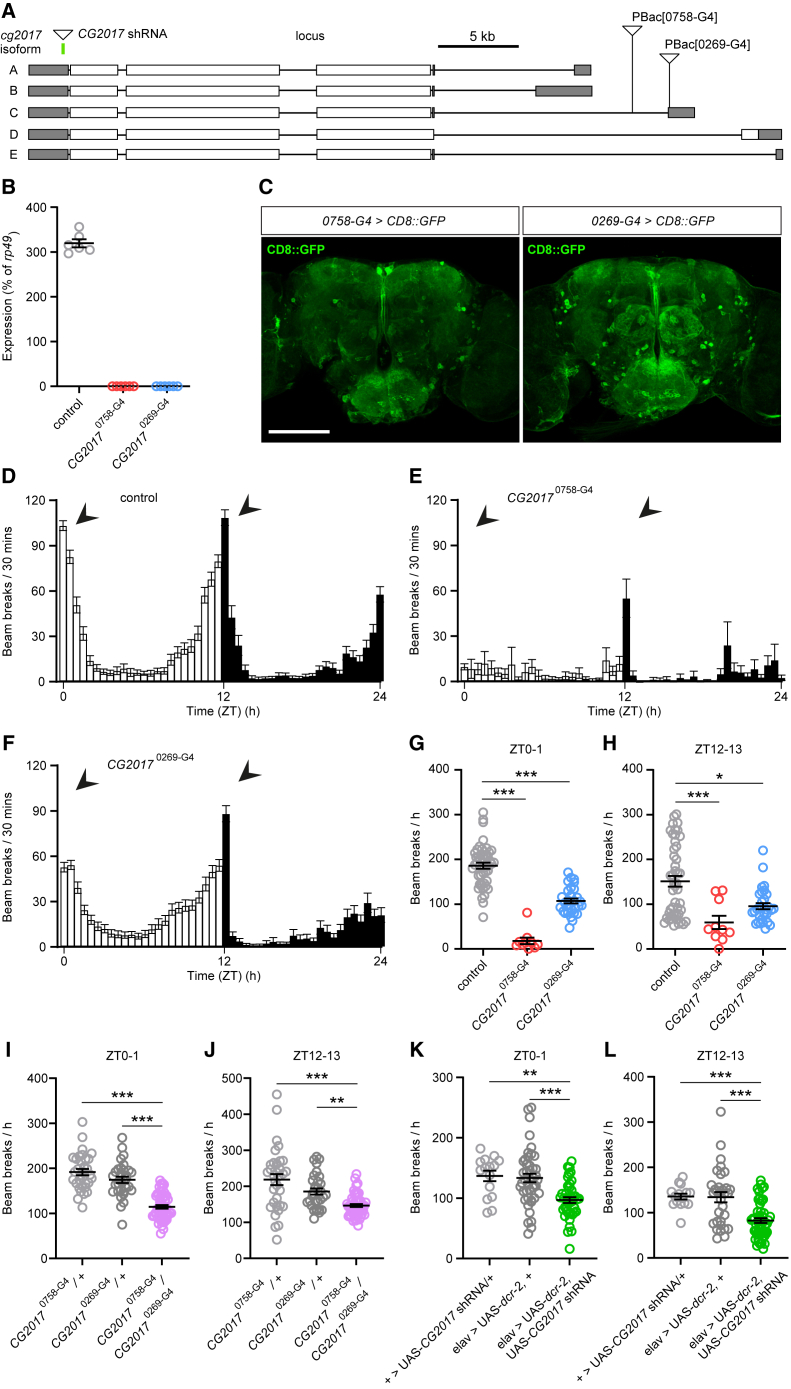
Suppressing expression of the *Drosophila GTPBP1/2* homolog *CG2017* reduces locomotor activity (A) Schematic of the *CG2017* locus. Gray regions: 3′ (left) and 5′ (right) UTRs. White regions: coding exons. Positions of the two piggyBac elements studied in this work are noted. Both leave the 5′ UTRs of CG2017 isoforms A and B intact, while likely impacting transcription from upstream 5′ UTRs (isoforms C–E). (B) qPCR analysis of expression of isoform C of *CG2017* in adult male iso31 controls and in *CG2017*^0^^758-G4^ and *CG2017*^0^^269-G4^ homozygotes. (C) Gal4 encoded within the Pbac [0758-G4] and Pbac [0269-G4] elements was used to report neural *CG2017* expression by driving transcription of membrane-targeted GFP (CD8::GFP). Scale bar, 100 μm. (D–F) Activity levels across a 12 h light:dark period in iso31 controls (D) and in *CG2017*^0758^^-G4^ (E) and *CG2017*^0^^269-G4^ (F) homozygotes. ZT: zeitgeber time. Arrows in (D) point to periods of peak activity in iso31 flies following lights-on and lights-off transitions. Arrows at equivalent positions in (E) and (F) illustrate changes in peak activity in *CG2017*^0^^758-G4^ and *CG2017*^0^^269-G4^ homozygotes. (G and H) Total beam breaks in iso31 control males, and in *CG2017*^0758^^-G4^ and *CG2017*^0^^269-G4^ homozygotes, measured between ZT0–1 (G) or ZT12–13 (H). Population sizes for (D)–(H) are as follows: Iso31, N = 47; *CG2017*^0758^^-G4^, N = 10; *CG2017*^0^^269-G4^, N = 31. (I and J) Total beam breaks in *CG2017*^0^^758-G4^/*CG2017*^0^^269-G4^ trans-heterozygous flies (N = 53) and heterozygote controls for each insertion (*CG2017*^0^^758-G4^/+, N = 33; *CG2017*^0^^269-G4^/+, N = 31), measured between ZT0–1 (I) or ZT12–13 (J). (K and L) Total beam break in flies co-expressing *CG2017* shRNA and Dicer-2 in neurons (*elav* > UAS-*CG2017* shRNA, UAS-*dcr-2*; N = 41) and controls (*elav* > UAS-*dcr-2*; N = 44; + > UAS-*CG2017* shRNA: N = 15) measured between ZT0–1 (K) or ZT12–13 (L). ^∗^p < 0.05, ^∗∗∗^p < 0.0005, Kruskal-Wallis test with Dunn’s post-hoc test (G, H, J, L) or one-way ANOVA with Tukey’s multiple comparisons test (I, K).

Finally, we aimed to test whether reducing expression of *GTPBP1* and *GTPBP2* homologs in an *in vivo* model system yielded phenotypes to similar those observed in individuals harboring *GTPBP1/2* variants. To do so we manipulated the expression of the *Drosophila GTPBP1* and *GTPBP2* ortholog, *CG2017*. Transcription of *CG2017* arises from five partially overlapping promoter regions (isoforms A–E; Figure 4A), and the CG2017 protein exhibits strong homology to GTPBP2 (48% identity, 66% similarity) and substantial homology to GTPBP1 (39% identity, 57% similarity) (https://flybase.org/reports/FBgn0037391; see supplemental information). We obtained transgenic fly lines harboring independent piggyBac transposable elements (*CG2017*^0^^758-G4^ and *CG2017*^0^^269-G4^) in, or close to, the 5′ promoter region of *CG2017* isoform C (Figure 4A). Insertion of such elements has the potential to disrupt *CG2017* transcription, mimicking loss-of-function (LoF). Indeed, using quantitative RT-PCR we found that homozygosity for *CG2017*^0^^758-G4^ and *CG2017*^0^^269-G4^ abolished detectable expression of *CG2017* isoform C (Figure 4B). The *CG2017*^0^^758-G4^ and *CG2017*^0^^269-G4^ elements encode the yeast transcriptional activator Gal4, which will thus be expressed under control of *CG2017* promoter/enhancer regions. By crossing flies with these insertions to a strain containing a fluorescent reporter under control of the Gal4 binding site (*UAS-CD8::GFP*), these lines can be used as reporters of *CG2017* transcription. Using this approach, we found *CG2017* transcripts from the isoform C promoter (and potentially the isoform D and E promoters) are expressed in a relatively restricted number of cells within the adult fly brain (Figure 4C). Projections from these neurons innervate numerous central neuropil regions previously shown to modulate movement or to be activated during movements, including the superior medial and lateral protocerebrum, the pars intercerebralis, and the gnathal ganglia.^21^^,^^22^^,^^23^ Since severe neurodevelopmental motor impairment is a core component of *GTPBP1/2*-related disorders in our cohort, we tested whether reduction in *CG2017* isoform C expression perturbed movement in *Drosophila*. Using the *Drosophila* Activity Monitor system,^20^ we found that homozygosity for the *CG2017*^0^^758-G4^ element profoundly reduced locomotor activity across the 24 h day/night cycle (Figures 4D and 4E) and strongly decreased the magnitude of startle responses to lights-on and lights-off transitions (times of peak locomotor activity in wild-type flies) (Figures 4G and 4H). Homozygotes for the *CG2017*^0^^269-G4^ insertion displayed an intermediate phenotype in which the pattern of crepuscular activity observed in wild-type flies was maintained but peak locomotor responses to lights-on and lights-off transitions were significantly reduced (Figures 4D and 4F–4H). Flies harboring trans-heterozygous combinations of the *CG2017*^0^^758-G4^ and *CG2017*^0^^269-G4^ elements also exhibited significant reductions in peak locomotor responses to lights-on/lights-off transitions compared to flies heterozygous for either element alone (Figures 4I and 4J). In addition, we utilized cell-specific RNAi-based knockdown of *CG2017* to test whether CG2017 acts in neurons to regulate movement. We expressed a short hairpin RNA (shRNA) transgene predicted to target the 3′ UTR region from all *CG2017* isoforms (Figure 4A) under control of the pan-neuronal driver *elav*-Gal4, in concert with a UAS-*dicer-2* (UAS-*dcr-2*) transgene to enhance the potential efficacy of knockdown.^24^^,^^25^ Similarly to flies harboring LoF alleles in *CG2017*, neuronal expression of *CG2017* shRNA reduced peak locomotor responses to lights-on/lights-off transitions compared to transgene and driver-alone controls (Figures 4K and 4L).

Collectively, these data demonstrate a conserved role for GTPBP1/2 and CG2017 in promoting normal movements and suggest a key role for these proteins in neurons.

## Discussion

The clinical syndrome associated with bi-allelic *GTPBP1* or *GTPBP2* variants can be defined as a complex syndromic neurodevelopmental disorder associated with severe neurodegeneration, epilepsy, choreiform movement disorder, and ectodermal abnormalities, with pathognomonic craniofacial features. Genetic defects of GTP-binding proteins may result in abnormal processes of ribosomal homeostasis and translation from mRNA to proteins^3^; however, exact mechanisms have been so far unidentified.

*GTPBP1* variants have not been reported before in humans, and so there is no known Mendelian disease associated with genetic defects in *GTPBP1*. The *GTPBP2*-related NDDs have been recently described in a limited number of families with clinical manifestations that are almost identical to *GTPBP1*. The first reported family was found to carry a homozygous (splicing) variant in *GTPBP2* and associated MRI findings of NBIA. Clinical features associated with this first family included developmental delay and ID, but some individuals showed social interaction and an ataxic gait, and additional findings including tremor and dystonia.^4^ After this initial report, the condition associated with bi-allelic *GTPBP2* variants was termed “Jaber-Elahi syndrome” (MIM: 617988). However, only one out five *GTPBP2*-variant families showed NBIA-related imaging abnormalities and marked neurological involvement (compared to the first family) characterized by profound impairment of developmental milestones and no achievement of any significant motor control.^5^^,^^6^ Thus, clinical delineation and genotype-phenotype correlations, as well as underlying disease mechanisms, of *GTPBP2*-related NDDs are yet to be fully defined. In this study, we used WES, WGS, and targeted sequencing to investigate 20 individuals from 16 families affected with undiagnosed syndromic NDDs associated with severe and progressive brain/cerebellar atrophy. We identified homozygous missense and truncating variants in *GTPBP1* and homozygous missense, truncating, and splicing variants in *GTPBP2* associated with a strikingly identical clinical presentation. Shared phenotypic features, representing the cardinal characteristics of this syndrome, combine congenital and/or postnatal microcephaly, profound neurodevelopmental impairment (across multiple domains), and distinctive craniofacial features, including a coarse face, high temporal and frontal hairline, bitemporal narrowing, full cheeks, thick protruding lips and macroglossia. In individuals with variants in *GTPBP1* and *GTPBP2*, distinct signs of abnormal ectodermal development such thin sparse hair, sparse eyebrows and eyelashes, abnormal nails, hand/feet anomalies, abnormal dentition (hypodontia), and abnormalities of skin pigmentation and sweat glands were all variably present. Neurological features associated with this syndrome included axial hypotonia, marked motor impairment (with absent motor milestones in all individuals), severe to profound cognitive impairment with absence of speech, frequent refractory seizures (with onset in the first year of life), variable sensorineural hearing and visual impairment (with evidence of retinal dysfunction in some affected individuals), severe and progressive spastic tetraparesis (predominantly at lower limbs), distinct hyperkinetic movements (including myoclonic jerks and peculiar distal choreoathetoid movements mostly involving the hands), and CNS hypoplasia and/or atrophy (mainly affecting the cerebellum). Based on previously reported individuals with *GTPBP2* variants and the present cohort (that includes both *GTPBP1*- and *GTPBP2*-variant carriers exhibiting an identical phenotypic presentation), we propose to define this condition as *G*tpbp1/2-*r*elated *e*ctodermal *n*euro*d*evelopmental (GREND) syndrome.

Notably, the core features associated with GREND syndrome appear to be phenotypically recognizable, making this a distinct clinical entity. However, a combination of ectodermal craniofacial features and similar (progressive) neurological features (including dystonia and extrapyramidal signs) can be observed in individuals affected with Woodhouse-Sakati syndrome (WDSKS [MIM:241080]) that carry bi-allelic pathogenic variants in the *DCAF17* gene (MIM: 612515). Interestingly, *DCAF17* has been previously implicated in NBIA-related phenotypes, and variants in this gene have been associated with dysregulated ribosome biogenesis and abnormalities in mRNA processing and transport.^26^

To assess the functional impact of the identified variants, available fibroblast cell lines from affected individuals were used to perform semi-qPCR and western blot analyses, which identified significant reduction of both mRNA and protein levels, respectively, suggesting a LoF mechanism of the variants.

Notably, the *GTPBP1* and the *GTPBP2* genes encode poorly characterized GTP-binding proteins that are highly expressed in the CNS of humans and mice.^2^^,^^27^ In mice, homozygous loss of GTPBP2 in combination with a second variant in a brain-specific arginine tRNA gene leads to a severe neurological disorder with severe motor deficits and progressive ataxia.^28^ Although the exact role of GTPBP1/2 in the human brain is not yet fully elucidated, experimental and animal model studies highlighted the function of these GTPases in regulating ribosome homeostasis and preventing abnormal translation events, such as ribosomal stalling.^1^ Ribosome-interacting factors can be critical for different quality-control mechanisms such as avoiding abnormal translation arrests by targeting its products for degradation.^28^ Ribosome dysfunction and/or deficiency can affect mRNA integrity and stability, and aberrant mRNAs may result in neuronal death and affecting physiological brain development.^29^

Aberrant mRNAs are produced by errors in mRNA maturation steps, and these factors recognize aberrant ribosome stalling and induce rapid degradation of aberrant polypeptides and mRNAs, thereby maintaining protein homeostasis and avoiding protein aggregation.^30^ Recently, GTPBP1 was also found to be required to relieve ribosome stalling and maintain neuronal homeostasis.^31^

However, using ribosome profiling, we did not observe any significant evidence of increased ribosome stalling or other translational defects in GTPBP2-deficient fibroblast cells. Previously, ribosome rescue by Gtpbp2 in complex with Pelota was observed in neurons of mice that lacked a specific isodecoder tRNA, but not in mice from other genetic backgrounds.^28^ Importantly, we cannot rule out a similar scenario in the affected individuals, as ribosome stalling in human neurons lacking GTPBP2 may be cell-type specific and/or only occur under specific cellular stresses not captured in our assay. Nonetheless, consistent with a causative role for LoF variants in *GTPBP1* or *GTPBP2* in this syndrome, reduced expression of the *GTPBP1*/*2* homolog *CG2017* in *Drosophila* resulted in marked locomotor defects, which is consistent with the severe motor impairment of the affected individuals from this cohort.

Collectively, our findings add insights into the phenotypic consequences of bi-allelic LoF genetic variants in both GTPBP1 and GTPBP2, highlighting that “GREND syndrome” can result from the loss of either homologous translational GTPase in humans. Further studies will be needed to reveal mechanistic insights into how abnormal brain development occurs in the context of these “ribosomopathies” and to assess the role of aberrant translational control on CNS function in humans as well as across different species.

## Data and code availability

The accession numbers for the DNA variant data are LOVD: 0000946888, 0000946889, 0000946890, 0000946891, 0000946896, 0000946897, 0000946898, 0000946899, 0000946907, 0000946909, 0000946910, 0000946911, 0000946912, 0000946913, 0000946914. All analyzed data consist of individuals’ personal data and are stored according to the regulations of the institutions involved in this study. Anonymized data are available by request from the corresponding author.

## Consortia

The members of the SYNaPS Study Group are Michael G. Hannah, Enrico Bugiardini, Enrico Bertini, Yamna Kriouile, Mohamed El-Khorassani, Mhammed Aguennouz, Stanislav Groppa, Blagovesta M. Karashova, Jatinder S. Goraya, Tipu Sultan, Daniela Avdjieva, Hadil Kathom, Radka Tincheva, Selina Banu, Pierangelo Veggiotti, Alberto Verrotti, Marcello Lanari, Salvatore Savasta, Alfons Macaya, Barbara Garavaglia, Eugenia Borgione, Savvas Papacostas, Michail Vikelis, Viorica Chelban, Rauan Kaiyrzhanov, Andrea Cortese, Roisin Sullivan, Eleni Z. Papanicolaou, Efthymios Dardiotis, Shazia Maqbool, Shahnaz Ibrahim, Salman Kirmani, Nuzhat N. Rana, Osama Atawneh, Shen-Yang Lim, Gian V. Zuccotti, Gian L. Marseglia, Susanna Esposito, Farooq Shaikh, Paola Cogo, Giovanni Corsello, Salvatore Mangano, Rosaria Nardello, Donato Mangano, Annarita Scardamaglia, George Koutsis, Carmela Scuderi, Eugenia Borgione, Pietro Ferrara, Giovanna Morello, Massimo Zollo, Roberto Berni-Canani, Luigi M. Terracciano, Antonio Sisto, Sandra Di Fabio, Federica Strano, Giovanna Scorrano, Saverio Di Bella, Ludovica Di Francesco, Ganieva Manizha, Maksud Isrofilov, Ulviyya Guliyeva, Kamran Salayev, Samson Khachatryan, Georgia Xiromerisiou, Cleanthe Spanaki, Chiara Fiorillo, Michele Iacomino, Eugenio Gaudio, Francina Munell, Antonella Gagliano, Farida Jan, Roberto Chimenz, Eloisa Gitto, Lorenzo Iughetti, Gabriella Di Rosa, Mohamad Maghnie, Massimo Pettoello-Mantovani, Neerja Gupta, Madhulika Kabra, Hanene Benrhouma, Meriem Tazir, Gabriella Bottone, Giovanni Farello, Maurizio Delvecchio, Giulio Di-Donato, Makram Obeid, Sophia Bakhtadze, Nebal W. Saadi, Michele Miraglia-Del-Giudice, Rita Maccarone, Maha S. Zaki, Chahnez C. Triki, Majdi Kara, Ehsan G. Karimiani, Ahmed M. Salih, Luca A. Ramenghi, Marco Seri, Giovanna Di-Falco, Luana Mandarà, Giuseppe Barrano, Maurizio Elisa, Enrico Cherubini, Francesca F. Operto, Mariella Valenzise, Antonino Cattaneo, Francesca Zazzeroni, Edoardo Alesse, Sara Matricardi, Faisal Zafar, Ehsan Ullah, Erum Afzal, Fatima Rahman, Muhammad M. Ahmed, Pasquale Parisi, Alberto Spalice, Maria De Filippo, Amelia Licari, Edoardo Trebbi, Ferdinando Romano, Gali Heimer, Issam Al-Khawaja, Fuad Al-Mutairi, Fowzan S. Alkuraya, Mie Rizig, Chingiz Shashkin, Nazira Zharkynbekova, and Kairgali Koneyev. The full list including titles and affiliations is available as supplemental information.

## Supplemental information

Document S1. Figures S1–S7 and supplemental methods

Table S1. GTPBP1 and GTPBP2 variants identified in this study

Table S2. Clinical features of patient with bi-allelic variants in GTPBP1 NM_004286.5 (light blue) or GTPBP2 NM_019096.6 (light gray)

Document S2. Article plus supplemental information
